# Antioxidative enzyme NAD(P)H quinone oxidoreductase 1 (NQO1) modulates the differentiation of Th17 cells by regulating ROS levels

**DOI:** 10.1371/journal.pone.0272090

**Published:** 2022-07-29

**Authors:** Kyoko Nishida-Tamehiro, Akihiro Kimura, Takeshi Tsubata, Satoru Takahashi, Harumi Suzuki

**Affiliations:** 1 Department of Immunology and Pathology, Research Center for Hepatitis and Immunology, Research Institute, National Center for Global Health and Medicine, Ichikawa-shi, Chiba, Japan; 2 Department of Immunology, Medical Research Institute, Tokyo Medical and Dental University, Bunkyo-ku, Tokyo, Japan; 3 Department of Anatomy and Embryology, Laboratory Animal Resource Center in Transborder Medical Research Center, Faculty of Medicine, University of Tsukuba, Tsukuba-shi, Ibaraki, Japan; University of Texas at San Antonio, UNITED STATES

## Abstract

NAD(P)H quinone oxidoreductase 1 (NQO1) is a flavoprotein that catalyzes two-electron reduction of quinone to hydroquinone by using nicotinamide adenine dinucleotide (NADPH), and functions as a scavenger for reactive oxygen species (ROS). The function of NQO1 in the immune response is not well known. In the present study, we demonstrated that *Nqo1*-deficient T cells exhibited reduced induction of T helper 17 cells (Th17) *in vitro* during Th17(23)- and Th17(β)- skewing conditions. *Nqo1*-deficient mice showed ameliorated symptoms in a Th17-dependent autoimmune Experimental autoimmune encephalomyelitis (EAE) model. Impaired Th17-differentiation was caused by overproduction of the immunosuppressive cytokine, IL-10. Increased IL-10 production in *Nqo1*-deficient Th17 cells was associated with elevated intracellular Reactive oxygen species (ROS) levels. Furthermore, overproduction of IL-10 in Th17 (β) cells was responsible for the ROS-dependent increase of c-*avian musculoaponeurotic fibrosarcoma* (c*-maf*) expression, despite the lack of dependency of c-maf in Th17(23) cells. Taken together, the results reveal a novel role of NQO1 in promoting Th17 development through the suppression of ROS mediated IL-10 production.

## Introduction

T helper 17 cells (Th17) are defined as interleukin-17 (IL-17)-producing cells which are important for the defense against extracellular bacteria. They are also involved in the pathogenesis of various autoimmune diseases [1]. Th17 cells could be subdivided into inflammatory Th17 (described as Th17(23)) cells and suppressive Th17 (described as Th17(β)) cells, resulting from exposure to IL-6 + IL1β + IL-23, or IL-6 + transforming growth factor-beta (TGFβ) [2, 3], respectively. The Th17(23) cells are involved in the pathogenesis of rheumatoid arthritis, multiple sclerosis, and inflammatory bowel disease, whereas Th17(β) cells can suppress disease symptoms by IL-10 production [4].

IL-10 is a robust immunosuppressive cytokine, which inhibits the development, growth, and function of various immune cells [5–8]. It also regulates the mucosal immune response against gut microbes to promote the homeostasis of mucosal immunity. Various innate and adaptive immune cells such as Th1, Th2, Th17(β), Tr1, Treg, dendritic cells, and macrophages have a capacity to produce IL-10. Transcription of the *Il-10* gene is regulated by various cell-specific transcription factors, such as signal transducer and activator of transcription 4 (STAT4), nuclear factor of activated T-cells (NFAT), and B-lymphocyte-induced maturation protein 1 (Blimp1) in Th1 cells, STAT6 and basic leucine zipper ATF-Like transcription factor (BATF) in Th2 cells, STAT3 and aryl hydrocarbon receptor (AhR) and Blimp1 in Th17 cells, and Blimp1 in regulatory T (Treg) cells [9–12]. The transcription factor, c-avian musculoaponeurotic fibrosarcoma (c-maf), is involved in many T cell types [10, 12]. Expression of c-maf is strongly induced in Th17(β), iTreg, and Type 1 regulatory cells (Tr1), but not in Th17(23) cells [13]. The transcription factor c-maf plays multiple roles in Th17 cells. For example, it induces IL-10 production, promotes RAR-related orphan receptor gamma (RORγt) expression, and inhibits IL-22 and interferon gamma (IFNγ) expression [14].

Reactive oxygen species (ROS) are generated as by-products of various metabolic processes and are highly reactive, causing harm to cells. However, low concentrations of ROS are recognized as an important second messenger in various signal transduction pathways. In T cells, T cell receptor (TCR) stimulation, as well as the metabolic shift upon differentiation and activation, induces ROS generation to regulate function and differentiation. Accordingly, ROS is reported to modulate Th17 differentiation [15–18]. However, the impacts of ROS on Th17 cell differentiation are controversial and unclear.

Intracellular concentrations of ROS are strictly regulated and homeostatically maintained by several antioxidants and antioxidative enzymes, such as glutathione and superoxide dismutase (SOD) [19]. Among antioxidative enzymes, NAD(P)H quinone oxidoreductase 1 (NQO1) has unique characteristics to scavenge ROS by multiple mechanisms. Besides direct reduction of ROS [20], NQO1 indirectly reduces ROS via reducing antioxidants such as Vitamin E [21]. Additionally, NQO1 reduces quinone to hydroquinone without formation of semiquinone, which induce ROS production, resulting in the suppression of ROS production [21]. The expression of NQO1 is induced by drug metabolisms and stress reactions by transcription factors, such as NF-E2-related factor 2 (Nrf2) and AhR [21, 22]. We have studied the function of NQO1 in the immune system and discovered a novel function of NQO1 in macrophages and dendritic epidermal T cells [23, 24]. Involvement of some antioxidative enzymes such as Gpx in Th17 differentiation has been reported [18], however, analysis of the function of NQO1, a unique antioxidative enzyme would shed light on comprehensive understanding of the effect of ROS signaling on Th17 differentiation.

In the present study, we focused on the function of NQO1 on Th17 differentiation and found that NQO1 modulates Th17 differentiation. NQO1 was highly expressed in Th17 cells and inhibited ROS accumulation. In the absence of NQO1 in T cells, intracellular ROS was increased upon activation and accumulated ROS led to overproduction of IL-10, resulting in the suppression of Th17 differentiation. In Th17(β) cells, ROS-dependent overproduction of IL-10 was c-maf-dependent, whereas it was independent of c-maf in Th17 (23) cells. Collectively, we identified a novel role of NQO1 in Th17 cell differentiation and demonstrated the involvement of NQO1 in acquired immunity.

## Materials & methods

### Mice

C57BL/6J mice were purchased from SLC Japan (Shizuoka, Japan). *Recombination activating gene2* (*RAG2*)-deficient mice were purchased from Jackson Laboratory. *Nqo1* KO mice (C57BL/ 6J background) were provided by A.K. Jaiswal (University of Maryland, Baltimore) [25]. c-*maf Flox* mice (C57BL/6J background) were provided by S. Takahashi (University of Tsukuba, Japan). *Il-10 venus* reporter mice were provided by K. Takeda (Osaka University, Japan) [26]. All mice were housed under specific pathogen-free conditions in our animal facility. Anesthesia was used each time to reduce pain for mice. All mice experiments (6–12 week of age) were performed by skilled staffs under protocols approved by the Animal Care and Use Committee of the National Center for Global Health Medicine Research Institute (No.21050).

### Induction of active and adoptive EAE and disease analysis

Four to five female B6J WT or *Nqo1*-deficient mice were injected subcutaneously in the back with 200 μg CFA-MOG mixture which premixed MOG (35–55) (BEX) with an equal volume of complete Freund’s adjuvant (DIFCO) containing of H37RA (BD Biosciences, San Jose, CA, USA). These mice were injected with 200 μg/ml Pertussis toxin i.p. (Merck Millipore, Burlington, MA, USA) in PBS on days 0 and 2. To examine infiltrating cells into the central nervous system, spinal cord and brain were collected from mice perfused with physiological saline containing heparin under anesthesia. For adoptive transfer EAE, CD4^+^ T cells were harvested from donor mice at peak of disease (days 21). These cells were cultured in the presence of MOG antigen under the Th17 (23) condition for 3 days. These cells were injected into recipient *Rag2*-deficient mice i.v. at 10 million cells /mice. Clinical score follows; 0: normal, 1: Partially limp tail, 2: Complete limp tail, 3: Partial hind limb paralysis, 4: Complete hind limb paralysis, 5: Moribund state (euthanasia), 6: death. Clinical score and animal health were monitored once every two days. Mice were euthanized at end of experiments (at 23–30 days), or at the humane endpoints defined by > 20% loss in maximal body weight, or appearance of general paralysis symptoms.

### ELISA

The concentrations of mouse IL-10, IL-17A, IL-17AF and IL-22 from supernatants of the culture media were measured by means of ELISA according to the manufacturer’s instructions (eBioscience, San Diego, CA, USA).

### Quantitative mRNA analysis

Total RNA was extracted from cells by using the RNeasy Mini kit (Qiagen) and reverse transcribed with ReverTra Ace qPCR RT Master Mix (TOYOBO, Japan). Real-time PCR was performed with the SYBR Premix ExTaq (TaKaRa, Japan) and StepOne Real-Time PCR System (Thermo Fisher, USA). All RT-PCR experiments were normalized with the internal control *Gapdh*. Relative mRNA levels were calculated using a standard curve of a total cDNA dilution series starting with an arbitrary number and using the CFX384 software. The following primers were used: *Gapdh*, sense 5′- AACTTTGGCATTGTGGAAGG, antisense 5′-GGATGCAGGGATGATGTTCT; *Nqo1*, sense 5’-TAGTCCCAGTTAGATGGCATCC, antisense 5′-CTCGGCCATTGTTTACTTTG; c*-maf*, sense 5’-TGGAGATCTCCTGCTTGAGG, antisense 5’- TGACACCTTACAAAACCCGGA; *Catalase*, sense 5’-AGCGACCAGATGAAGCAGTG, antisense 5’-TCCGCTCTCTGTCAAAGTGTG; *Sod1*, sense 5’-AACCAGTTGTGTTGTCAGGAC, antisense 5’-CCACCATGTTTCTTAGAGTGAGG; *Sod2*, sense 5’-CAGACCTGCCTTACGACTATGG, antisense 5’-CTCGGTGGCGTTGAGATTGTT; *Gpx1*, sense 5’-AGTCCACCGTGTATGCCTTCT, antisense 5’-GAGACGCGACATTCTCAATGA; *Gpx2*, sense 5’-GCCTCAAGTATGTCCGACCTG, antisense 5’-GGAGAACGGGTCATCATAAGGG. The amplification reaction was performed as follows: 30 seconds at 95 °C, followed by 40 cycles of denaturation (5 s at 95 °C), primer annealing, and extension (30 s at 60 °C), followed by melting curve analysis.

### Naïve CD4^+^ T cell preparation and T cell differentiation

Naïve CD4^+^ T cells were enriched from splenocytes using negative selection for depletion of B220, CD8α, CD11b, CD11c, CD25, CD44, TER119, Ly6G, NK1.1 positive cells. And then, CD62L^+^ cells were isolated from B220^-^, CD8α ^-^, CD11b^-^, CD11c^-^, CD25^-^, CD44^-^, TER119^-^, Ly6G^-^, NK1.1^-^ fraction using CD62L MicroBeads and MACS MS column and MACS separator (Miltenyi Biotec). This fraction was CD4^+^ CD62L^hi^ naïve T cells and was confirmed to be > 90% pure population by flow cytometry before use.

### Cell culture

For Th17 cell differentiation, 2.5 x 10^5^ isolated naïve CD4^+^ T cells were cultured with plate bound 2 μg/ml anti-CD3 (Biolegend, USA) and 2 μg/ml soluble anti-CD28 (Biolegend,) for 3 days. The following cytokine mixes were added to skew each subset.

Th17(β): 2 ng/ml TGF-β (R&D systems, Minneapolis, MN, USA), 20 ng/ml IL-6 (Biolegend,).

Th17(23): 20 ng/ml IL-1β (Biolegend,), 20 ng/ml IL-6 (Biolegend,), 25 ng/ml IL-23 (R&D systems, USA).

### Cell analysis

For cell-surface staining and intracellular staining, the following Abs were used: anti-IL-17A (Tc11-18H10.1), anti-IL-10R (1B1.3a), anti-RORγt (B2D), anti-CD4 (RM4-5), anti-CD8 (53–6.7), anti-c-maf (Sym0F1), and anti-Foxp3 (150D). All Abs were purchased from BioLegend or eBioscience.

The transcription staining was performed with Foxp3 staining kit (eBioscience), according to the manufacturer’s protocol. For, cytokine analysis, cultured cells were stimulated phorbol 12-myristate 13-acetate (PMA), ionomysin, and brefeldin A (BFA) for 4 h at 37°C and fixed with 4% paraformaldehyde for 30 minutes, permeabilized with 1x permeabilized buffer (eBioscience).

FACS analysis was performed using a FACS Canto II (BD Biosciences) and the data were analyzed with Flow Jo software, version 10.2.

### Analysis of ROS

For the depletion of ROS, NAC (N-acetyl cysteine) was dissolved in DDW at 1 M and added into the cell culture media at a final concentration of 5 mM.

For detection of oxidative stress, three types of ROS detection probes, DCFDA (ENZO life science, USA), DHE (Cayman, USA), and mitochondrial ROS detection kit (Cayman), were used.

### Statistical analysis

Data were analyzed with Graphpad Prism software. Statistical signified were assessed by an unpaired Student’s t test or one-way ANOVA with multiple comparisons. P-values <0.05 were considered statistically significant. Data are presented as means ±SE.

## Results

### NQO1 promotes Th17 polarization *in vitro*

**Fig 1:**
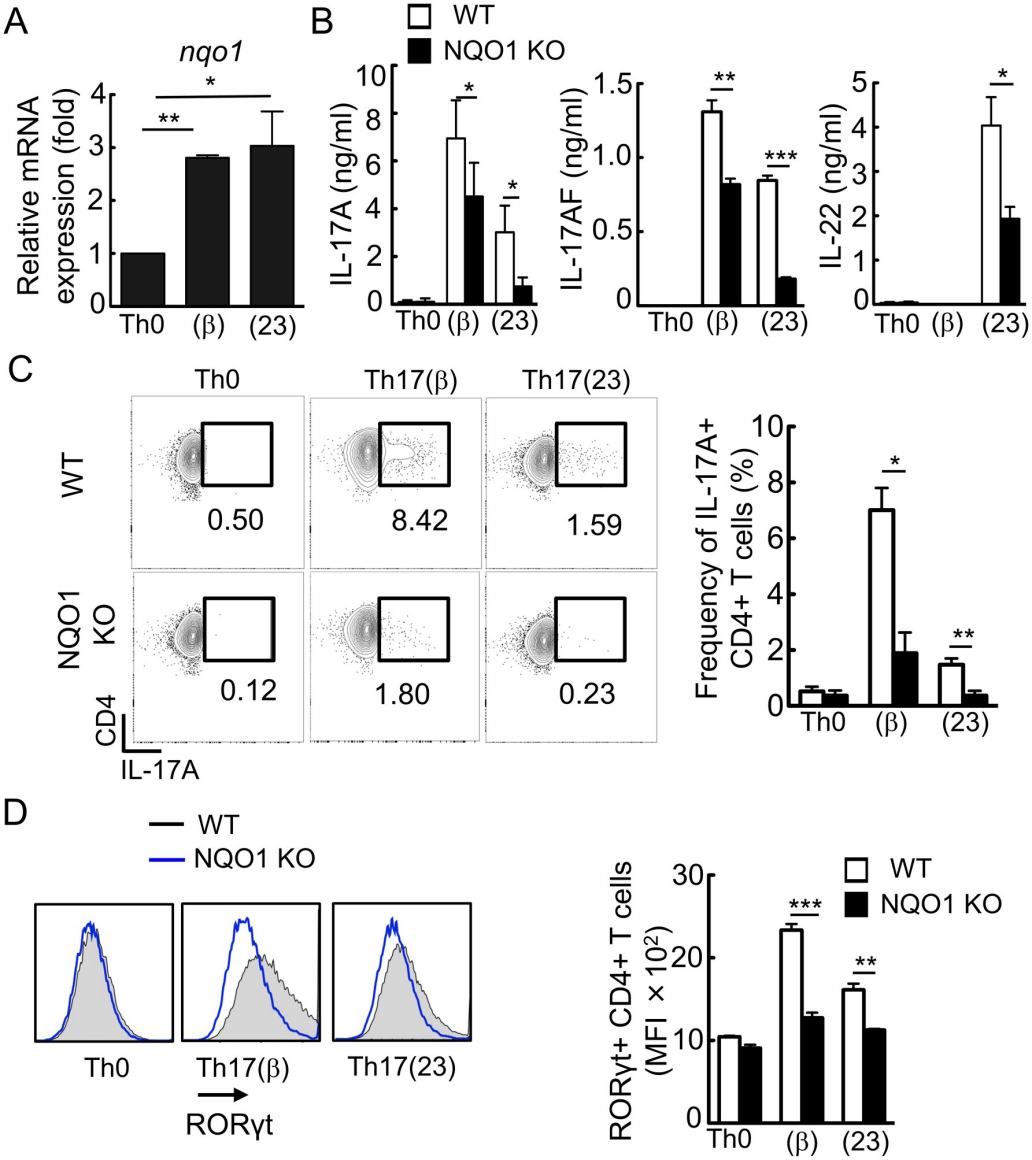
NQO1 promotes Th17 differentiation *in vitro*. Isolated CD4^+^ CD62L^hi^ naïve T cells were stimulated with anti-CD3 (2 μg/ml) and anti-CD28 (2 μg/ml) antibodies and cultured with Th17(β) (stimulated with IL-6 +TGFβ) and Th17(23) (stimulated with IL-1β +IL-6 +IL-23) skewing conditions for 3 days. (A) Real-time PCR analysis of *Nqo1* gene expression in WT (n = 3). (B) IL-17A and IL-22 production in the cell culture supernatant was measured by ELISA. (n = 5) (C) On days 3, the differentiated cells were stimulated with PMA, Ionomycin, and BFA for 4 h. The plot indicates frequencies of CD4^+^ IL-17A^+^ T cells (n = 3). (D) Histogram showing internal staining of CD4^+^ RORγt^+^ expression (n = 3). Each graph indicates the mean ± SE, One way ANOVA.

First, we analyzed the expression of *Nqo1* mRNA in induced Th17 cells, which were differentiated from naïve CD4^+^CD62L^hi^ T cells *in vitro*. As shown in Fig 1A, *Nqo1* was highly expressed in Th17(β) cells (induced with TGFβ + IL-6) and Th17(23) cells (induced with IL-1β + IL-6 + IL-23) compared with Th0 cells (TCR stimulation without cytokines). We examined the effect of NQO1 on Th17 differentiation *in vitro*. Induction of both Th17(β) and Th17(23) from naïve CD4^+^ T cells after 3 days of culture was significantly suppressed in *Nqo1*-deficinent T cells as determined by internal IL-17A staining and secretion of IL-17A and IL-17AF into the culture media (Fig 1B and 1C). Proliferation and absolute number of cultured Th17 cells were comparable between *Nqo1*-deficient and WT cells (S1 Fig). The production of IL-22, which is typically produced by Th17 cells, was also reduced in *Nqo1*-deficient Th17(23) cells (Fig 1B, right panel). Expression of Foxp3 was not changed in these cells (S1 Fig). Consistently, induction of RORγt expression, an essential transcription factor for Th17 differentiation, was markedly inhibited in *Nqo1*-deficient T cells (Fig 1D). These data indicated the involvement of NQO1 in Th17 development.

### NQO1 deficiency upregulated the production of IL-10

**Fig 2:**
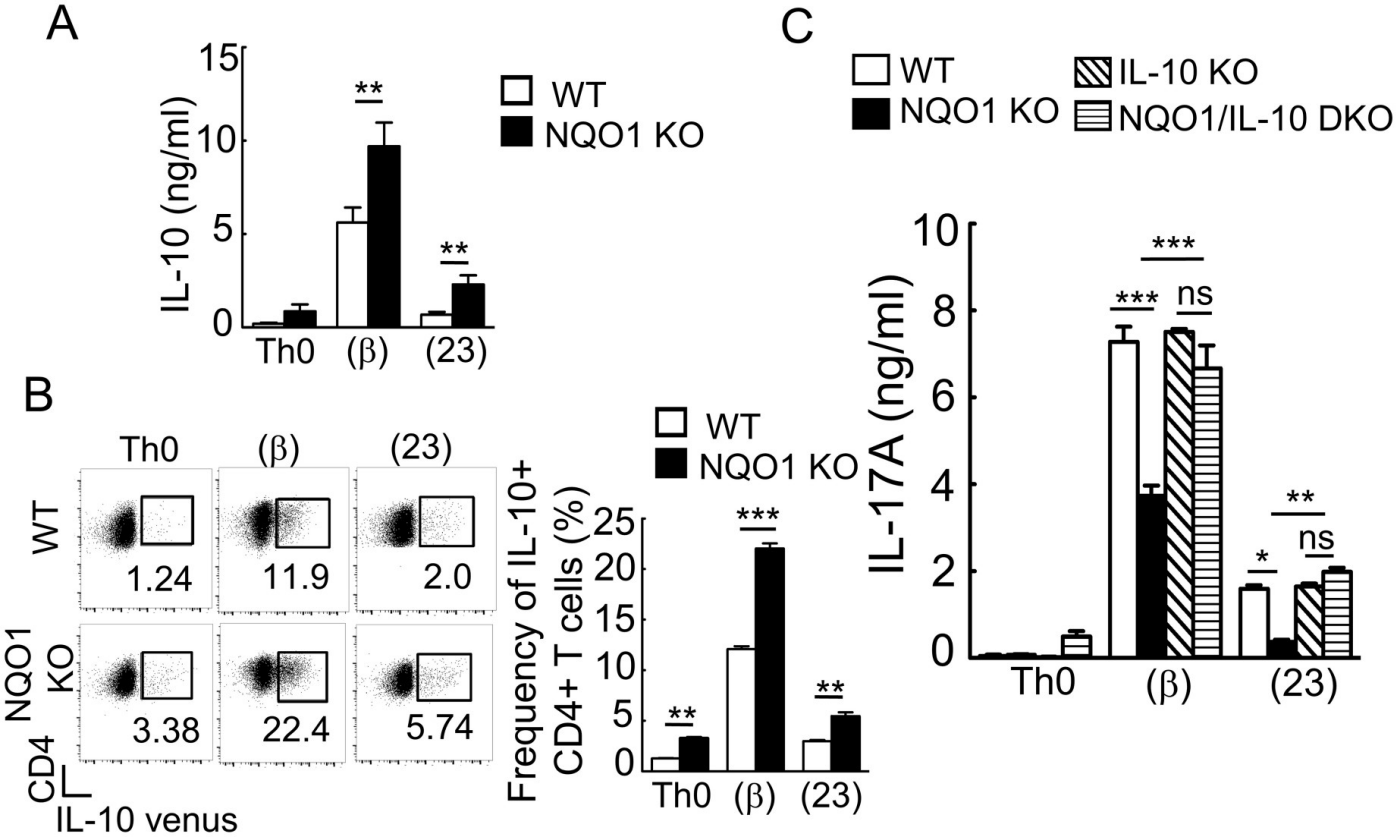
NQO1 promotes Th17 differentiation by inhibiting IL-10 production. (A, C) Purified naïve CD4^+^ T cells from WT, *Nqo1* KO, *Il-10* KO, or *Nqo1*/*Il-10* double knock-out were stimulated with anti-CD3/CD28 antibody in each differentiation conditions for 3 days. Cytokines secreted into the cell culture supernatant were measured by ELISA (n = 4–8). (B) Naïve CD4^+^ T cells from *Il-10 venus* reporter mice, and *Nqo1* KO/*Il-10 venus* reporter mice were differentiated into Th17 (β) or Th17 (23) cells (n = 3). The ratio of IL-10 expressing CD4^+^ cells cultured under each condition for 3 days was determined. Data are representative of three independent experiments. Graphs indicate the mean ± SE, One way ANOVA.

IL-10 is known to suppress IL-17 production and RORγt expression, resulting in the inhibition of Th17 differentiation [5–7]. We hypothesized that *Nqo1* deficiency promotes IL-10 production which inhibits Th17 differentiation. As shown in Fig 2A, the amount of IL-10 produced under Th17(β)- and Th17(23)-inducing culture conditions was significantly increased in *Nqo1*-deficient cells. The increased frequency of IL-10 producing cells in *Nqo1*-deficient CD4^+^ T cells was confirmed by flow cytometry utilizing IL-10 reporter mice (Fig 2B) [26].

To confirm the dependency of IL-10 on NQO1-mediated suppression of Th17 differentiation, we generated double knock-out mice that were deficient in *Nqo1* and *Il-10*. As shown in Fig 2C, the introduction of *Il-10* deficiency into *Nqo1* knock-out T cells successfully restored reduced IL-17A production in both Th17(β) and Th17(23) cells. These results indicated that *Nqo1* deficiency enhanced IL-10 production and concomitantly suppressed Th17 differentiation.

### NQO1 plays a crucial role in Th17 differentiation *in vivo*

**Fig 3:**
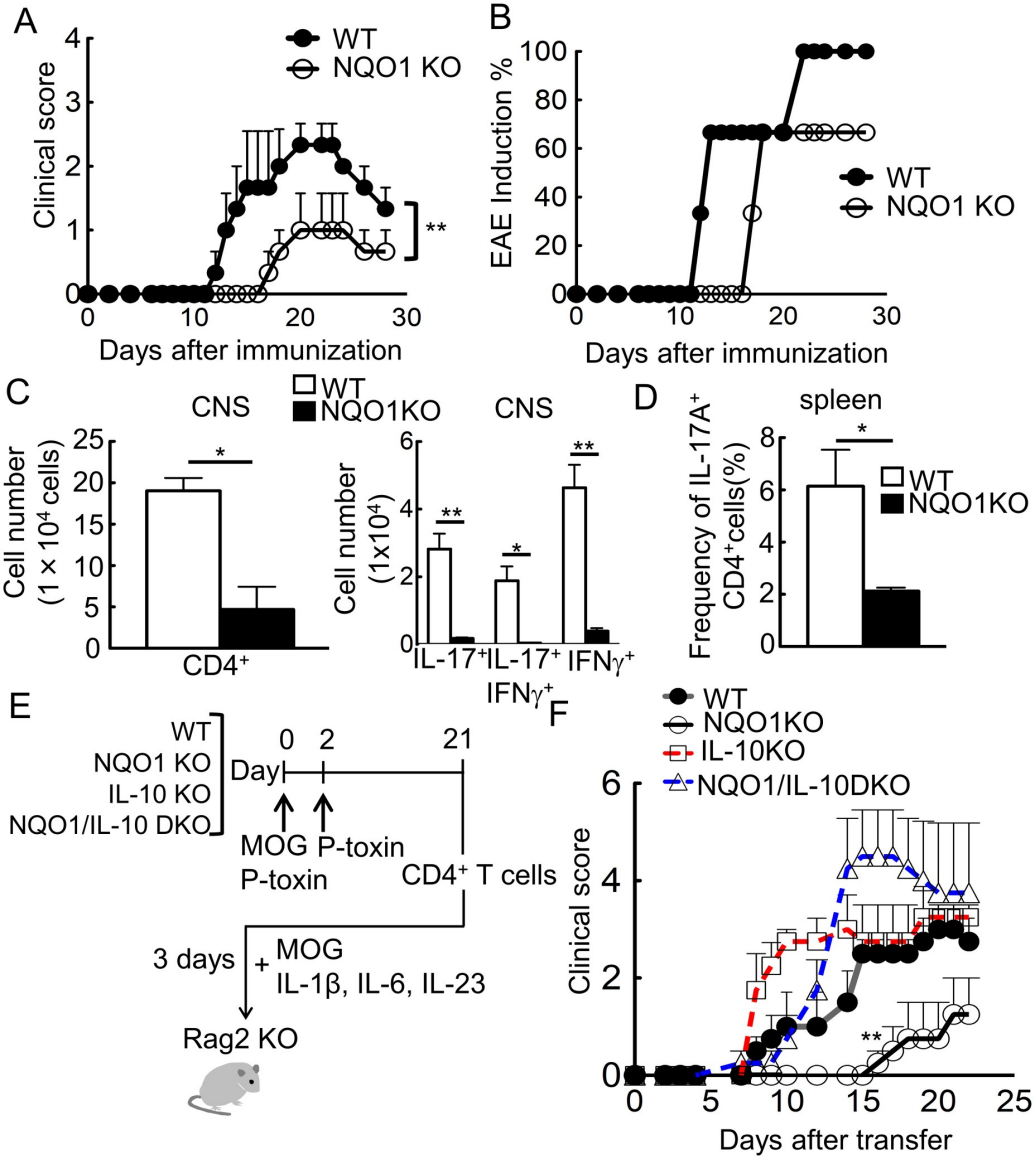
*Nqo1* deficiency ameliorates EAE severity depend on IL-10. (A, B) The graph shows clinical scores and induction ratio for EAE in WT, *Nqo1* KO mice after immunization with a MOG-CFA mixture and p-toxin. Mice were sacrificed under anesthesia at end of experiment (n = 4). (C) Absolute numbers of CNS infiltrating cells were analyzed. Total CD4^+^ cells are presented in left panel and IL-17 expression CD4^+^ cells are shown in right panel (n = 4). (D) Splenocytes from immunized mice were re-stimulated with 50 μM MOG peptide for 72 h. Frequencies of CD4^+^ IL-17^+^, IFNγ^+^ cells (%) were measured by FACS (n = 4). (E, F) The graph indicates clinical scores for adoptive transfer EAE in recipient mice (n = 5). Mice were sacrificed under anesthesia at 23 days (end of experiment) or at the humane endpoints. Data are representative of three independent experiments. Graphs indicate the mean ± SE. (A-C) Unpaired student T-test, (D-F) One way ANOVA.

As has been reported, development of T cells in *Nqo1*-deficienct mice was normal [27], therefore the frequency of IL-17^+^, RORgt^+^, or Foxp3^+^ CD4^+^ T cells in spleen and mesenteric lymph node was not altered in *Nqo1*-deficienct mice (S2 Fig). To investigate the effect of NQO1 on Th17 induction *in vivo*, *Nqo1*-deficient mice were examined by an autoimmune Experimental autoimmune encephalomyelitis (EAE) model, in which pathogenesis is dependent upon Th17 cells. As we expected, *Nqo1*-deficient mice exhibited a significant decrease in disease severity (Fig 3A), which is consistent with reduced Th17 induction observed *in vitro* (Fig 1). In addition, the incidence of active EAE induction in *Nqo1*-deficient mice was nearly 60%, despite the 100% incidence in wild-type mice (Fig 3B). Then, infiltrated cells in the central nervous system (CNS) were analyzed. As shown in Fig 3C, absolute number of CNS infiltrating CD4^+^ T cells, as well as IL-17 producing, IFNγ producing, and IL-17/IFNγ double producing CD4^+^ T cells were strongly reduced in *Nqo1*-deficient animals (Fig 3C). In addition, IL-17 production of splenic CD4^+^ T cells from these EAE mice by re-stimulation with MOG peptide *in vitro* was significantly reduced in *Nqo1*-deficient mice (Fig 3D). Collectively, these results suggested the involvement of NQO1 in Th17 differentiation *in vivo*.

To examine the T cell intrinsic function of NQO1, we next performed an adoptive transfer EAE experiment. CD4^+^ T cells from the spleens of EAE-induced mice were stimulated with MOG antigen and Th17(23) -skewing cytokines *in vitro*. These activated CD4^+^ T cells were transferred into *Rag2*-deficient mice (Fig 3E). As shown in Fig 3F, the adoptive transfer EAE experiments revealed essentially the same results of active EAE. Furthermore, these ameliorated symptoms in *Nqo1*-deficient mice were dependent upon IL-10, because double knock-out mice for NQO1 and IL-10 restored the clinical scores and disease incidence to that of wild-type mice (Fig 3F). That is, transfer of *Nqo1*-deficient CD4^+^ T cells exhibited a reduced clinical score, whereas *Nqo1*/*Il-10* DKO exhibited a recovered score. Collectively, these results indicated that NQO1 regulates the Th17-related immune response *in vivo*.

### Accumulation of ROS in *Nqo1*-deficient T cells augments IL-10 production

**Fig 4:**
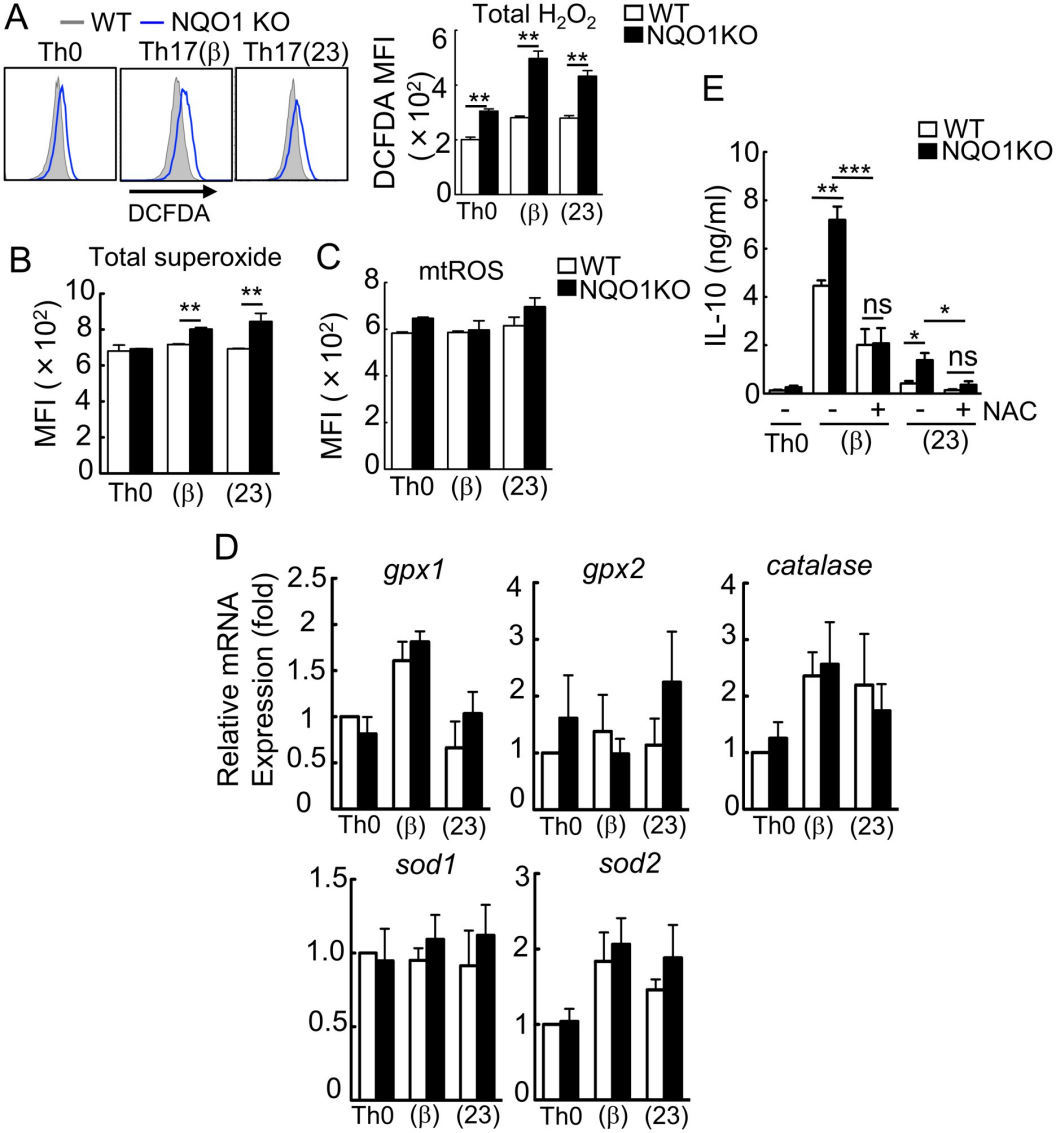
NQO1 inhibits IL-10 production by regulating ROS. (A-C) Naïve CD4^+^ T cells from *Nqo1* KO and WT mice were differentiated into Th0, Th17(β), and Th17(23) cells with the indicated cytokines for 1 day. Cells were loaded with the ROS indicator, DCFDA, DHE, or mtROS detecting reagent and the fluorescence intensities were detected by flow cytometry (n = 3). (D) IL-10 secretion in the cell culture supernatant with or without NAC (5 mM) as measured by ELISA (n = 4). (D) The expressions of a lot of antioxidative enzymes were measured by RT-PCR (n = 4). These graphs indicate the mean ± SE, One way ANOVA.

The amount of ROS is controlled by antioxidants and antioxidative enzymes, such as SOD, Gpx, and NQO1, to maintain an optimum concentration [19–21]. Therefore, the absence of NQO1 would increase ROS accumulation. We examined the total H_2_O_2_ levels in *Nqo1*-deficient Th17 cells in 24 hours after activation by 2’,7’-dichlorofluorescin diacetate (DCFDA) staining. At the same time, total superoxide (O_2_^-·^) levels were examined by dihydroethidium (DHE) staining. As expected, we observed increased total ROS in *Nqo1*-deficient Th17 cells (Fig 4A and 4B). On the contrary, mitochondrial ROS (mtROS) levels, measured by Mitochondrial ROS detection kit (Cayman) was unchanged between wild-type and *Nqo1*-deficient T cells (Fig 4C). Therefore, accumulated ROS in *Nqo1*-deficient Th17 cells should be cytosolic ROS. To exclude the possibility of compensatory increase of other antioxidative enzymes, we measured mRNA expression of various other antioxidative enzymes in *Nqo1*-deficient T cells, resulting in unaltered expressions (Fig 4D).

Since ROS induces IL-10 production in human and murine macrophages [28, 29], we hypothesized that the accumulated ROS induces augmented IL-10 production in *Nqo1*-deficient Th17 cells. Thus, we tested the effect of the antioxidant, N-acetyl-cysteine (NAC), which inhibits ROS accumulation on the production of IL-10 in *Nqo1*-deficient Th17 cells. As shown in Fig 4E, NAC treatment decreased IL-10 production in *Nqo1*-deficient T cells compared to that of the wild-type T cells. Additionally, addition of NAC partially recovered the reduced IL-17 production in *Nqo1*-deficient Th17 cells (S3 Fig). Collectively, these results indicated that ROS accumulation in *Nqo1*-deficient T cells is responsible for the increased production of IL-10.

### Increased IL-10 production is dependent on c-maf in *Nqo1*-deficient Th17(β) cells, but not in Th17(23) cells

**Fig 5:**
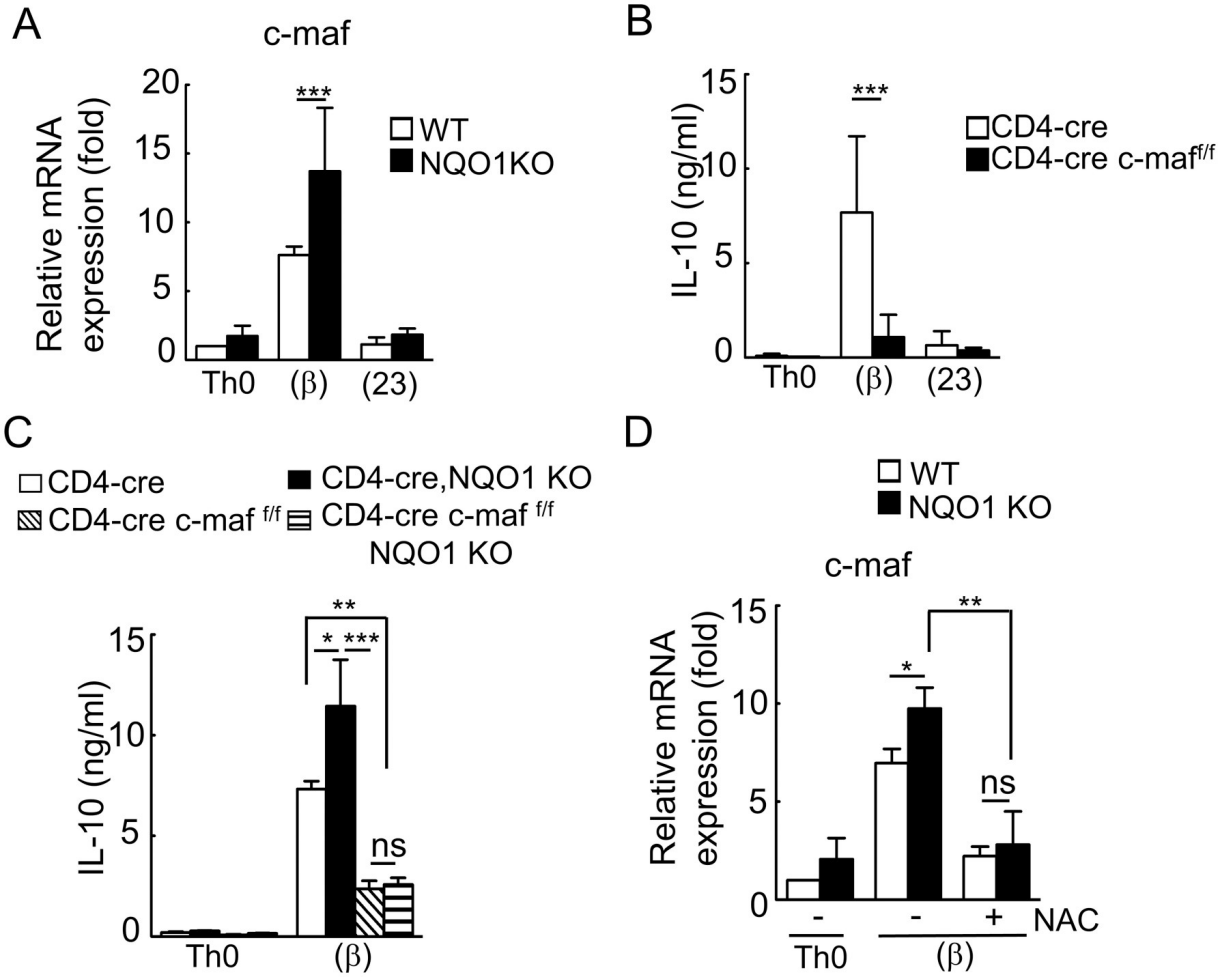
NQO1 inhibits IL-10 production by controlling c-maf upregulation by ROS in Th17(β) cells. (A) Isolated naïve CD4^+^ T cells from *Nqo1*KO or WT mice stimulated with Th0, Th17(β), and Th17(23) skewing cytokine conditions for 3 days. The expression of *c-maf* was determined by RT-PCR (n = 5). (B) Naïve CD4^+^ T cells from CD4^*cre/+*^ or c-*maf*^*Flox/Flox*^, CD4^*cre/+*^ mice were differentiated into Th0, Th17(β), or Th17(23) cells for 3 days. Secreted IL-10 in the cell culture supernatant was measured by ELISA (n = 4). (C) Naïve CD4^+^ T cells from CD4^*cre/+*^ or *Nqo1* KO, CD4^*cre/+*^ or c-*maf*^*Flox/Flox*^, CD4^*cre/+*^ or *Nqo1* KO, c-*maf*^*Flox/Flox*^, CD4^*cre/+*^ mice were cultured in Th17(β) skewing conditions for 3 days. The secretion of IL-10 in the supernatant was measured by ELISA (n = 5). (D) c-*maf* gene expression with or without the antioxidant NAC (5 mM) was measured by RT-PCR (n = 4). Graphs indicate the mean ± SE, One way ANOVA.

Transcriptional regulation of IL-10 gene has been extensively studied and the leucine zipper transcription factor, c-maf, is known to play a pivotal role in IL-10 gene transcription in CD4^+^ T cells [8, 9, 11], which is reduced by depletion of c-maf [30]. Therefore, we next focused on the expression of c-*maf* in *Nqo1*-deficient T cells. As shown in Fig 5A, the expression of c-*maf* was markedly induced in Th17(β) cells, whereas *Nqo1* deficiency accelerated its expression. Consistent with previous reports [13], c-*maf* was not expressed in Th17(23) cells and its expression was not increased even in the absence of *Nqo1*. Therefore, we determined whether c-*maf*-induced IL-10 expression was evident in Th17(β) cells. Consistent with a previous report [30], the production of IL-10 was decreased in c-*maf*-deficient Th17(β) cells (Fig 5B). From these results, we hypothesized that increased c-*maf* expression is responsible for IL-10 overproduction in *Nqo1*-deficient Th17(β) cells. To test this idea, we established *Nqo1* and c-*maf* (*Nqo1* KO, *Cd4*^cre/+^, c-*maf* ^F/F^) double knock-out mice. As shown in Fig 5C, c-*maf* deficiency in *Nqo1*-deficient T cells successfully canceled the increased IL-10 production observed in *Nqo1*-deficient Th17(β) cells. From these results, increased IL-10 production in *Nqo1*-deficient Th17(β) cells was dependent on c-maf expression. Next, we examined the relationship between c-maf and ROS, because both are required for the overexpression of IL-10 in *Nqo1*-deficient Th17(β) cells. We determined the effect of NAC on c-maf induction in Th17(β) cells. As shown in Fig 5D, NAC treatment successfully canceled increased induction of c-*maf* in *Nqo1*-deficient Th17(β) cells. Collectively, in Th17(β) cells, *Nqo1* deficiency caused ROS accumulation to increase c-*maf* expression, leading to enhanced transcription of IL-10. In contrast, in the case of Th17(23) cells, ROS-induced IL-10 overproduction occurred in a c-maf-independent manner.

## Discussion

NQO1 functions as a ROS scavenger and its expression is induced by various environmental stimuli [20]. When effector T cells differentiate from naïve T cells, an enormous amount of ROS is generated by stimulation with TCR and cytokines such as IL-6 [31]. Indeed, ROS was accumulated in Th17(β), Th17(23) cells, however, *Nqo1* was induced concomitantly (Fig 1A), resulted in depletion of excess amount of ROS (Fig 4A and 4B). Upon stimulation, elevated ROS augments c-maf expression to accelerate IL-10 production resulting in the suppression of Th17 differentiation in Th17(β) cells. On the other hand, the same TCR/cytokine-stimulation simultaneously upregulates expression of NQO1, which counteract this ROS/c-maf/IL-10 axis by scavenging ROS. NQO1 acts as an upstream negative regulator against c-maf, and thus it may contribute to accelerate or maintain Th17-mediated inflammation.

IL-10 is a suppressive cytokine and is capable of inhibiting differentiation and function of various immune effector cells. A lot of studies reported that IL-10 deficient mice showed enhanced Th17 responses *in vivo* [6, 32, 33]. Accordingly, we observed accelerated EAE responses in IL-10 deficient mice (Fig 3F). However, IL-10-deficient Th17 cells did not exhibit enhanced IL-17 production in Th17-inducing cultures *in vitro* (Fig 2C). These unexpected results indicated that the amount of IL-10 produced by wild-type Th17 cells under these conditions was not sufficient to suppress Th17 differentiation. As a matter of fact, Wu et al. reported the IL-10-independent Th17 differentiation from naive CD4 T^+^ cells *in vitro* [34], although Zhang et al. reported discrepant IL-10-dependent *in vitro* Th17 differentiation in similar condition [35], indicating that *in vitro* results could be sensitive for detailed experimental conditions. Because expression levels of IL-10 receptor were not different between wild-type and *Nqo1* deficient Th17 cells (S4 Fig), substantial increase of IL-10 production in *Nqo1*-deficient Th17 cells may exceed the threshold to suppress Th17 differentiation.

We demonstrated that the accumulation of ROS in cells lacking NQO1 suppressed Th17 differentiation. Our observation is consistent with that of a previous report in which the depletion of the antioxidative enzyme, *Gpx1* (*Glutathione peroxidase 1*), resulted in ROS accumulation and suppression of Th17(β) differentiation [18]. Anti-oxidative enzymes play important roles in the regulation of Th17 differentiation, because the deficiency of two different antioxidative enzymes demonstrated the same phenotype. In the present study, we demonstrated that ROS-induced Th17 suppression was responsible for the overproduction of IL-10.

ROS can be measured as two different types, total (intracellular) ROS and mitochondrial ROS (mtROS). Total ROS represents sum of cytosolic ROS and mtROS. Although the effects of ROS on Th17 differentiation have been controversial, discrimination of cytosolic ROS and mtROS may solve this confusion. Zhi et al. reported that increased mtROS in *immediate early response gene X-1* (*Iex-1*)-deficient cells accelerated Th17(β) differentiation [15]. In contrast, two independent groups reported that suppressed production of cytosoilc ROS in NADPH oxidase 2 (NOX2)-deficient cells accelerated Th17(β) differentiation [16, 17]. Our results demonstrated that increased cytosolic ROS in *Nqo1*-deficient cells suppressed differentiation of both Th17(β) and Th17(23) cells. From these observations, mtROS promotes Th17 differentiation, whereas it is inhibited by cytosolic ROS, indicating that the effect of ROS may depend on the species and intracellular localization.

Interestingly, mtROS levels were not changed in *Nqo1*-deficient Th17 cells (Fig 4C), although total ROS levels was increased (Fig 4A and 4B). More than 90% of ROS is generated from mitochondria, therefore ROS generation in mitochondria should be more strictly controlled than the one in the cytosol. Indeed, several antioxidative enzymes such as SOD2, thioredoxin-2, and peroxiredoxin-3 are exclusively expressed in mitochondria. Since NQO1 is dominantly expressed in cytosol, depletion of *Nqo1* may have little effect on mtROS generation.

Low concentrations of ROS play an important role in various signal transduction pathways, such as TCR dependent activation and differentiation [36]. In Th17 cells, it was reported that ROS regulate IL-17A production [37]. We demonstrated that ROS is also involved in the IL-10 production in Th17(β) and Th17(23) cells (Fig 4E). Although ROS-dependent IL-10 production was reported in macrophages [28, 29], we firstly demonstrated the existence of the same pathway in T cells. Furthermore, we demonstrated that this ROS-induced IL-10 overproduction in Th17(β) cells was dependent on c-maf (Fig 5C and 5D), which is a central IL-10 transcription factor.

Ordinary Th17(23) cells do not produce IL-10 [1, 3], however, *Nqo1*-deficient Th17(23) cells abnormally produced small amounts of IL-10 (Fig 2A and 2B), which was dependent on ROS (Fig 4E). Although ROS-induced IL-10 production was dependent on c-maf in Th17(β) cells (Fig 5C and 5D), it was not the case in Th17(23) cells, because c-*maf* was not expressed in Th17(23) cells (Fig 5A) [13]. Therefore, there must be an alternative c-maf-independent pathway for ROS-induced IL-10 production. Synergistic stimulation with IL-6 and TGFβ induces vigorous c-maf expression, which is dependent on STAT3 activation [8]. Because ROS-dependent enhancement of STAT3 activation was reported in cancer cells [38], STAT3 may be involved in the ROS-dependent increase of c-*maf* expression. Akt was reported to be involved in c-maf-independent IL-10 production in Tr1 cells [39] and in ROS-induced IL-10 production in macrophage [28]. Because Akt can be activated not only by TCR signaling, but also by IL-23 signaling [40], Akt activation may be stronger in Th17(23) cells compared with Th17(β) cells. The stronger Akt activation in Th17(23) cells may replace the c-maf-dependent pathway for IL-10 production.

We showed that Th17-mediated inflammation was ameliorated in NQO1-deficient EAE model (Fig 3), indicating that inhibition of NQO1 by specific inhibitor such as dicoumarol could be a potential candidate for the therapeutic treatment against autoimmune diseases such as multiple sclerosis. Besides current strategy that aim to inhibit inflammatory cytokines, non-cytokine based approaches to suppress Th17 responses may be beneficial for the development of novel therapy for autoimmune diseases.

The relationship between Th17(β) and Th17(23) is still unclear and not well understood. These two subtypes may not represent the terminally differentiated lineage, but they may be mutually exchangeable depending on the cytokine environment. There could be intermediate type Th17 cells between Th17(β) and Th17(23). Indeed, some Treg-like suppressor Th17 cells (Th17(β)) existing in normal mucosa could acquire the proinflammatory phenotype (Th17(23)) upon exposure to IL-1β and IL-23 from inflamed environmental cells [1]. In other words, although Th17(β) exhibits a suppressive phenotype, it may instantly transform into an inflammatory phenotype upon environmental stimulation, and NQO1 may be a good candidate for the conversion. We demonstrated that even proinflammatory Th17(23) cells, which are involved in the pathogenesis of various autoimmune diseases, potentially produce the suppressive cytokine IL-10, through ROS accumulation. This observation may give rise to a possible new therapeutic approach for the treatment of autoimmune disease.

## Supporting information

S1 Fig There are no differences of proliferation, cell number and the frequency of Foxp3+ cells in cultured cells between WT and *Nqo1* deficiency. (A, B) Naïve CD4^+^ T cells from *Nqo1* KO and WT mice were differentiated into T0, Th17(β) and Th17(23) cells for 72 h. The absolute number of cultured cells was shown (A) (n = 3). Cells were loaded with cell tracker agent detecting cell proliferation and the fluorescence Intensities were detected by flow cytometry (B) (n = 3). (C) Expression of Foxp3 in the cells cultured with T0, Th17(β), Th17(23) each condition cytokine for 72 h (n = 4). These graphs indicate the mean ± SE, One way ANOVA.
(TIF)

S2 Fig The number and frequence of peripheral T cells. (A) Splenocytes from *Nqo1*^-/-^ and WT mice were stained for CD4 and CD8. The numbers indicated the percentages of cells (n = 4). (B) The gated CD4^+^ T cells were analyzed CD44 and CD62L expression in splenocytes (n = 4). (C) Splenocytes and lymphocytes from *Nqo1*KO and WT mice were stimulated with PMA, Ionomycin and BFA for 4 h and analyzed for IL-17A expressing CD4^+^ T cells by flow cytometry. Right panel showed the percentages of IL-17^+^ CD4^+^ T cells in mesenteric lymph node, and left panel displayed it in spleen from *Nqo1* KO and WT mice (n = 4). (D) The frequency of CD4^+^ RORγt^+^ T cells or CD4^+^ Foxp3^+^ T cells in splenocytes from *Nqo1* KO and WT mice in steady state (n = 4). These graphs indicate the mean ± SE, Unpaired T-test.
(TIF)

S3 Fig The effect of NAC treatment on IL-17 production. Naïve CD4^+^ T cells from WT and *Nqo1*-deficient mice were cultured under the Th17 conditions media for 72 h with or without 5 mM NAC. IL-17A production was measured by ELISA (n = 4). This graph indicates the mean ± SE, One way ANOVA.
(TIF)

S4 Fig The expression of IL-10R on the cell surface. Naïve CD4^+^ T cells from *Nqo1* deficiency and WT mice were cultured under the Th17 conditions media for 48 h. The graph showed expression of IL-10 receptor α (n = 4). This graph indicates the mean ± SE, One way ANOVA.
(TIF)

## References

[1] Wu B, Wan YS. Molecular control of pathogenic Th17 cells in autoimmune diseases. International Immunopharmacology. 2020;80. doi: 10.1016/j.intimp.2020.106187 . 31931372 PMC7031035

[2] Bauquet AT, Jin HL, Paterson AM, Mitsdoerffer M, Ho IC, Sharpe AH, et al. The costimulatory molecule ICOS regulates the expression of c-Maf and IL-21 in the development of follicular T helper cells and T-H-17 cells. Nature Immunology. 2009;10(2):167–175. doi: 10.1038/ni.1690 . 19098919 PMC2742982

[3] Lee Y, Awasthi A, Yosef N, Quintana FJ, Xiao S, Peters A, et al. Induction and molecular signature of pathogenic T(H)17 cells. Nature Immunology. 2012;13(10):991–999. doi: 10.1038/ni.2416 . 22961052 PMC3459594

[4] McGeachy MJ, Bak-Jensen KS, Chen Y, Tato CM, Blumenschein W, McClanahan T, et al. TGF-beta and IL-6 drive the production of IL-17 and IL-10 by T cells and restrain TH-17 cell-mediated pathology. Nature Immunology. 2007;8(12):1390–1397. doi: 10.1038/ni1539 . 17994024

[5] Heo YJ, Joo YB, Oh HJ, Park MK, Heo YM, Cho ML, et al. IL-10 suppresses Th17 cells and promotes regulatory T cells in the CD4(+) T cell population of rheumatoid arthritis patients. Immunology Letters. 2010;127(2):150–156. doi: 10.1016/j.imlet.2009.10.006 . 19895848

[6] Gu Y, Yang J, Ouyang X, Liu W, Li H, Bromberg J, et al. Interleukin 10 suppresses Th17 cytokines secreted by macrophages and T cells. European Journal of Immunology. 2008;38(7):1807–1813. doi: 10.1002/eji.200838331 . 18506885 PMC2733944

[7] Chaudhry A, Samstein RM, Treuting P, Liang YQ, Pils MC, Heinrich JM, et al. Interleukin-10 Signaling in Regulatory T Cells Is Required for Suppression of Th17 Cell-Mediated Inflammation. Immunity. 2011;34(4):566–578. doi: 10.1016/j.immuni.2011.03.018 . 21511185 PMC3088485

[8] Xu JN, Yang Y, Qiu GX, Lal G, Wu ZH, Levy DE, et al. c-Maf Regulates IL-10 Expression during Th17 Polarization. Journal of Immunology. 2009;182(10):6226–6236. doi: 10.4049/jimmunol.0900123 . 19414776 PMC2834209

[9] Pot C, Jin HL, Awasthi A, Liu SM, Lai CY, Madan R, et al. Cutting Edge: IL-27 Induces the Transcription Factor c-Maf, Cytokine IL-21, and the Costimulatory Receptor ICOS that Coordinately Act Together to Promote Differentiation of IL-10-Producing Tr1 Cells. Journal of Immunology. 2009;183(2):797–801. doi: 10.4049/jimmunol.0901233 . 19570826 PMC2768608

[10] Saraiva M, Christensen JR, Veldhoen M, Murphy TL, Murphy KM, O’Garra A. Interleukin-10 Production by Th1 Cells Requires Interleukin-12-Induced STAT4 Transcription Factor and ERK MAP Kinase Activation by High Antigen Dose. Immunity. 2009;31(2):209–219. doi: 10.1016/j.immuni.2009.05.012 . 19646904 PMC2791889

[11] Imbratta C, Hussein H, Andris F, Verdeil G. c-MAF, a Swiss Army Knife for Tolerance in Lymphocytes. Frontiers in Immunology. 2020;11. doi: 10.3389/fimmu.2020.00206 . 32117317 PMC7033575

[12] Chang KK, Liu LB, Jin LP, Zhang B, Mei J, Li H, et al. IL-27 triggers IL-10 production in Th17 cells via a c-Maf/ROR gamma t/Blimp-1 signal to promote the progression of endometriosis. Cell Death & Disease. 2017;8. doi: 10.1038/cddis.2017.95 . 28300844 PMC5386585

[13] Aschenbrenner D, Foglierini M, Jarrossay D, Hu D, Weiner HL, Kuchroo VK, et al. An immunoregulatory and tissue-residency program modulated by c-MAF in human T(H)17 cells. Nature Immunology. 2018;19(10):1126–1136. doi: 10.1038/s41590-018-0200-5 . 30201991 PMC6402560

[14] Rutz S, Noubade R, Eidenschenk C, Ota N, Zeng WW, Zheng Y, et al. Transcription factor c-Maf mediates the TGF-beta-dependent suppression of IL-22 production in T(H)17 cells. Nature Immunology. 2011;12(12):1238–1245. doi: 10.1038/ni.2134 . 22001828

[15] Zhi L, Ustyugova IV, Chen XY, Zhang Q, Wu MX. Enhanced Th17 Differentiation and Aggravated Arthritis in IEX-1-Deficient Mice by Mitochondrial Reactive Oxygen Species-Mediated Signaling. Journal of Immunology. 2012;189(4):1639–1647. doi: 10.4049/jimmunol.1200528 . 22798682 PMC3440304

[16] Tse HM, Thayer TC, Steele C, Cuda CM, Morel L, Piganelli JD, et al. NADPH Oxidase Deficiency Regulates Th Lineage Commitment and Modulates Autoimmunity. Journal of Immunology. 2010;185(9):5247–5258. doi: 10.4049/jimmunol.1001472 . 20881184 PMC3190397

[17] Lee K, Won HY, Bae MA, Hong JH, Hwang ES. Spontaneous and aging-dependent development of arthritis in NADPH oxidase 2 deficiency through altered differentiation of CD11b+and Th/Treg cells. Proceedings of the National Academy of Sciences of the United States of America. 2011;108(23):9548–9553. doi: 10.1073/pnas.1012645108 . 21593419 PMC3111328

[18] Won HY, Sohn JH, Min HJ, Lee K, Woo HA, Ho YS, et al. Glutathione Peroxidase 1 Deficiency Attenuates Allergen-Induced Airway Inflammation by Suppressing Th2 and Th17 Cell Development. Antioxidants & Redox Signaling. 2010;13(5):575–587. doi: 10.1089/ars.2009.2989 . 20367278

[19] Brien E, Sahiner MU, Sackesen C, Srzurum S, Kalayci O. Oxidative Stress and Antioxidant Defense. World Allergen Organ Journal. 2012;5(1):9–19. doi: 10.1097/WOX.0b013e3182439613 . 23268465 PMC3488923

[20] Siegel D, Gustafson DL, Dehn DL, Han JY, Boonchoong P, Berliner LJ, et al. NAD(P)H: quinone oxidoreductase 1: Role as a superoxide scavenger. Molecular Pharmacology. 2004;65(5):1238–1247. doi: 10.1124/mol.65.5.1238 . 15102952

[21] Ross D, Siegel D. Functions of NQO1 in Cellular Protection and CoQ(10) Metabolism and its Potential Role as a Redox Sensitive Molecular Switch. Frontiers in Physiology. 2017;8. doi: 10.3389/fphys.2017.00595 . 28883796 PMC5573868

[22] Ma Q, Kinneer K, Bi YY, Chan JY, Kan YW. Induction of murine NAD(P)H: quinone oxidoreductase by 2,3,7,8-tetrachlorodibenzo-p-dioxin requires the CNC (cap ’n’ collar) basic leucine zipper transcription factor Nrf2 (nuclear factor erythroid 2-related factor 2): cross-interaction between AhR (aryl hydrocarbon receptor) and Nrf2 signal transduction. Biochemical Journal. 2004;377:205–213. doi: 10.1042/BJ20031123 . 14510636 PMC1223846

[23] Kimura A, Kitajima M, Nishida K, Serada S, Fujimoto M, Naka T, et al. NQO1 inhibits the TLR-dependent production of selective cytokines by promoting I kappa B-(zeta) degradation. Journal of Experimental Medicine. 2018;215(8):2197–2209. doi: 10.1084/jem.20172024 . 29934320 PMC6080903

[24] Kitajima M, Kimura A, Suzuki H. Cutting Edge: Nqo1 Regulates Irritant Contact Hypersensitivity against Croton Oil through Maintenance of Dendritic Epidermal T Cells. Journal of Immunology. 2018;200(5):1555–1559. doi: 10.4049/jimmunol.1701389 . 29378915

[25] adjendirane V, Joseph P, Lee YH, Kimura S, Klein-Szanto AJP, Gonzalez FJ, et al. Disruption of the DT diaphorase (NQO1) gene in mice leads to increased menadione toxicity. Journal of Biological Chemistry. 1998;273(13):7382–7389. doi: 10.1074/jbc.273.13.7382 . 9516435

[26] Atarashi K, Tanoue T, Shima T, Imaoka A, Kuwahara T, Momose Y, et al. Induction of Colonic Regulatory T Cells by Indigenous Clostridium Species. Science. 2011;331(6015):337–341. doi: 10.1126/science.1198469 . 21205640 PMC3969237

[27] Iskander K, Li J, Han SH, Zheng B, Jaiswal AK. NQO1 and NQO2 regulation of humoral immunity and autoimmunity. Journal of Biological Chemistry. 2006;281(41):30917–30924. doi: 10.1074/jbc.M605809200 . 16905546

[28] Deng J, Wang XR, Qian F, Vogel S, Xiao L, Ranjan R, et al. Protective Role of Reactive Oxygen Species in Endotoxin-Induced Lung Inflammation through Modulation of IL-10 Expression. Journal of Immunology. 2012;188(11):5734–5740. doi: 10.4049/jimmunol.1101323 . 22547702 PMC3358534

[29] Kelly EK, Wang L, Ivashkiv LB. Calcium-Activated Pathways and Oxidative Burst Mediate Zymosan-Induced Signaling and IL-10 Production in Human Macrophages. Journal of Immunology. 2010;184(10):5545–5552. doi: 10.4049/jimmunol.0901293 . 20400701 PMC3016855

[30] Gabrysova L, Alvarez-Martinez M, Luisier R, Cox LS, Sodenkamp J, Hosking C, et al. c-Maf controls immune responses by regulating disease-specific gene networks and repressing IL-2 in CD4(+) T cells. Nature Immunology. 2018;19(5):497–507. doi: 10.1038/s41590-018-0083-5 . 29662170 PMC5988041

[31] Belikov AV, Schraven B, Simeoni L. T cells and reactive oxygen species. Journal of Biomedical Science. 2015;22. doi: 10.1186/s12929-015-0194-3 . 26471060 PMC4608155

[32] Sun MM, He C, Chen L, Yang WJ, Wu W, Chen FD, et al. ROR gamma t Represses IL-10 Production in Th17 Cells To Maintain Their Pathogenicity in Inducing Intestinal Inflammation. Journal of Immunology. 2019;202(1):79–92. doi: 10.4049/jimmunol.1701697 . 30478092 PMC6310078

[33] Wilke CM, Wang L, Wei S, Kryczek I, Huang E, Kao J, et al. Endogenous interleukin-10 constrains Th17 cells in patients with inflammatory bowel disease. Journal of Translational Medicine. 2011;9. doi: 10.1186/1479-5876-9-217 . 22176654 PMC3264534

[34] Wu XZ, Zhai K, Yi FS, Wang Z, Wang W, Wang Y, et al. IL-10 promotes malignant pleural effusion in mice by regulating T(H)1-and T(H)17-cell differentiation and migration. European Journal of Immunology. 2019;49(4):653–665. doi: 10.1002/eji.201847685 . 30695099

[35] Zhang RH, Li Q, Chuang PY, Lu GM, Liu RJ, Yang JJ, et al. Regulation of Pathogenic Th17 Cell Differentiation by IL-10 in the Development of Glomerulonephritis. American Journal of Pathology. 2013;183(2):402–412. doi: 10.1016/j.ajpath.2013.05.001 . 23747510 PMC3730759

[36] Lin WJ, Shen P, Song YQ, Huang Y, Tu SH. Reactive Oxygen Species in Autoimmune Cells: Function, Differentiation, and Metabolism. Frontiers in Immunology. 2021;12. doi: 10.3389/fimmu.2021.635021 . 33717180 PMC7946999

[37] Kim HR, Kim KW, Kim BM, Lee KA, Lee SH. N-acetyl-l-cysteine controls osteoclastogenesis through regulating Th17 differentiation and RANKL in rheumatoid arthritis. Korean Journal of Internal Medicine. 2019;34(1):210–219. doi: 10.3904/kjim.2016.329 . 28286938 PMC6325425

[38] Yoon S, Woo SU, Kang JH, Kim K, Kwon MH, Park S, et al. STAT3 transcriptional factor activated by reactive oxygen species induces IL6 in starvation-induced autophagy of cancer cells. Autophagy. 2010;6(8):1125–1138. doi: 10.4161/auto.6.8.13547 . 20930550

[39] Nadya NA, Tezuka H, Ohteki T, Matsuda S, Azuma M, Nagai S. PI3K-Akt pathway enhances the differentiation of interleukin-27-induced type 1 regulatory T cells. Immunology. 2017;152(3):507–516. doi: 10.1111/imm.12789 . 28685820 PMC5629445

[40] Zheng TT, Xu CC, Mao CM, Mou X, Wu F, Wang XF, et al. Increased Interleukin-23 in Hashimoto’s Thyroiditis Disease Induces Autophagy Suppression and Reactive Oxygen Species Accumulation. Frontiers in Immunology. 2018;9. doi: 10.3389/fimmu.2018.00096 . 29434604 PMC5796905

