# Supplementary figures and images for: Antioxidative enzyme NAD(P)H quinone oxidoreductase 1 (NQO1) modulates the differentiation of Th17 cells by regulating ROS levels
Source: PLoS One. 2022 Jul 29;17(7):e0272090. doi: 10.1371/journal.pone.0272090 (PMC9337673; doi:10.1371/journal.pone.0272090)

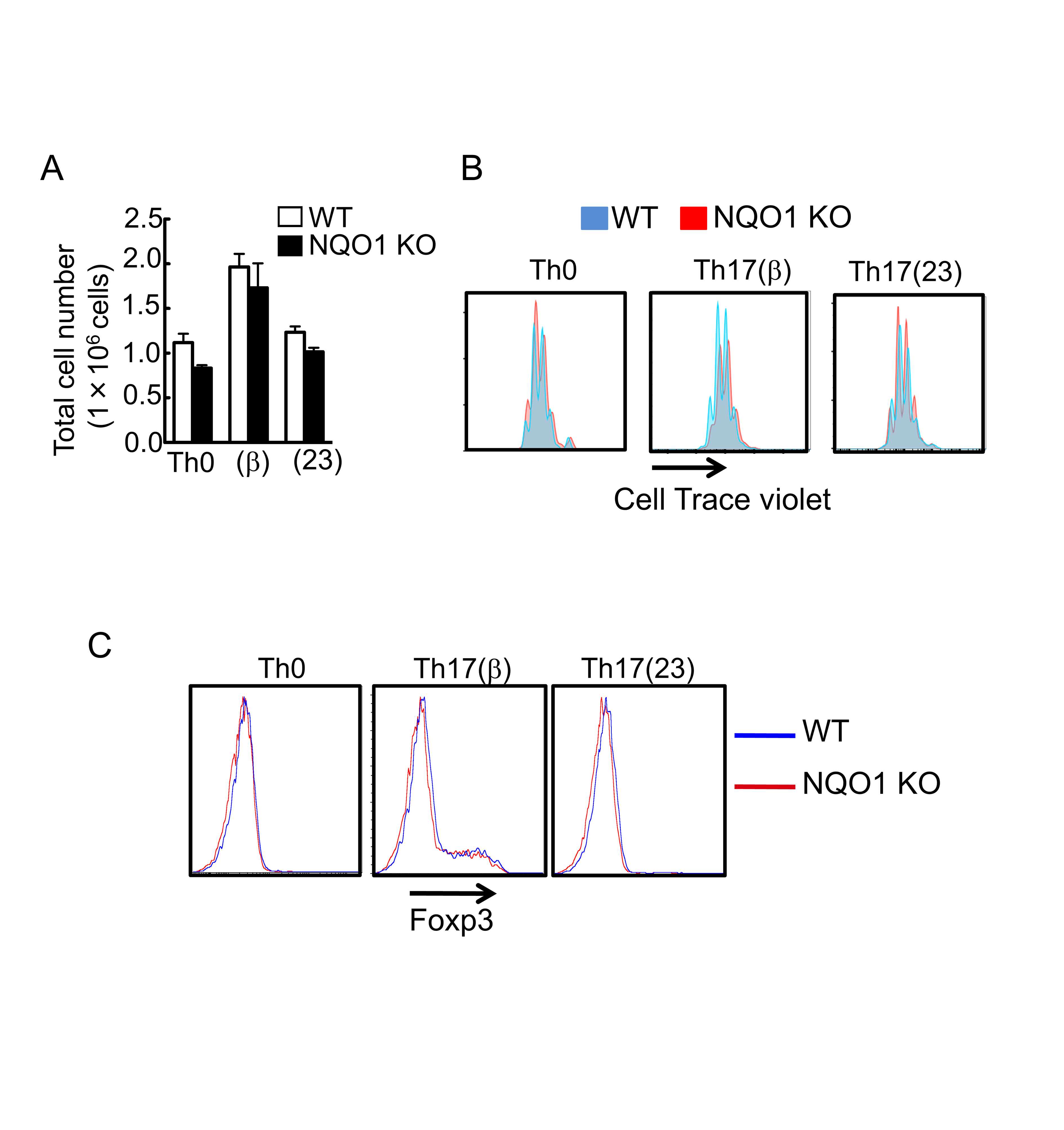

Supplement: S1 Fig — (A, B) Naïve CD4+ T cells from Nqo1 KO and WT mice were differentiated into T0, Th17(β) and Th17(23) cells for 72 h. The absolute number of cultured cells was shown (A) (n = 3). Cells were loaded with cell tracker agent detecting cell proliferation and the fluorescence Intensities were detected by flow cytometry (B) (n = 3). (C) Expression of Foxp3 in the cells cultured with T0, Th17(β), Th17(23) each condition cytokine for 72 h (n = 4). These graphs indicate the mean ± SE, One way ANOVA. (TIF) [file pone.0272090.s001.tif]

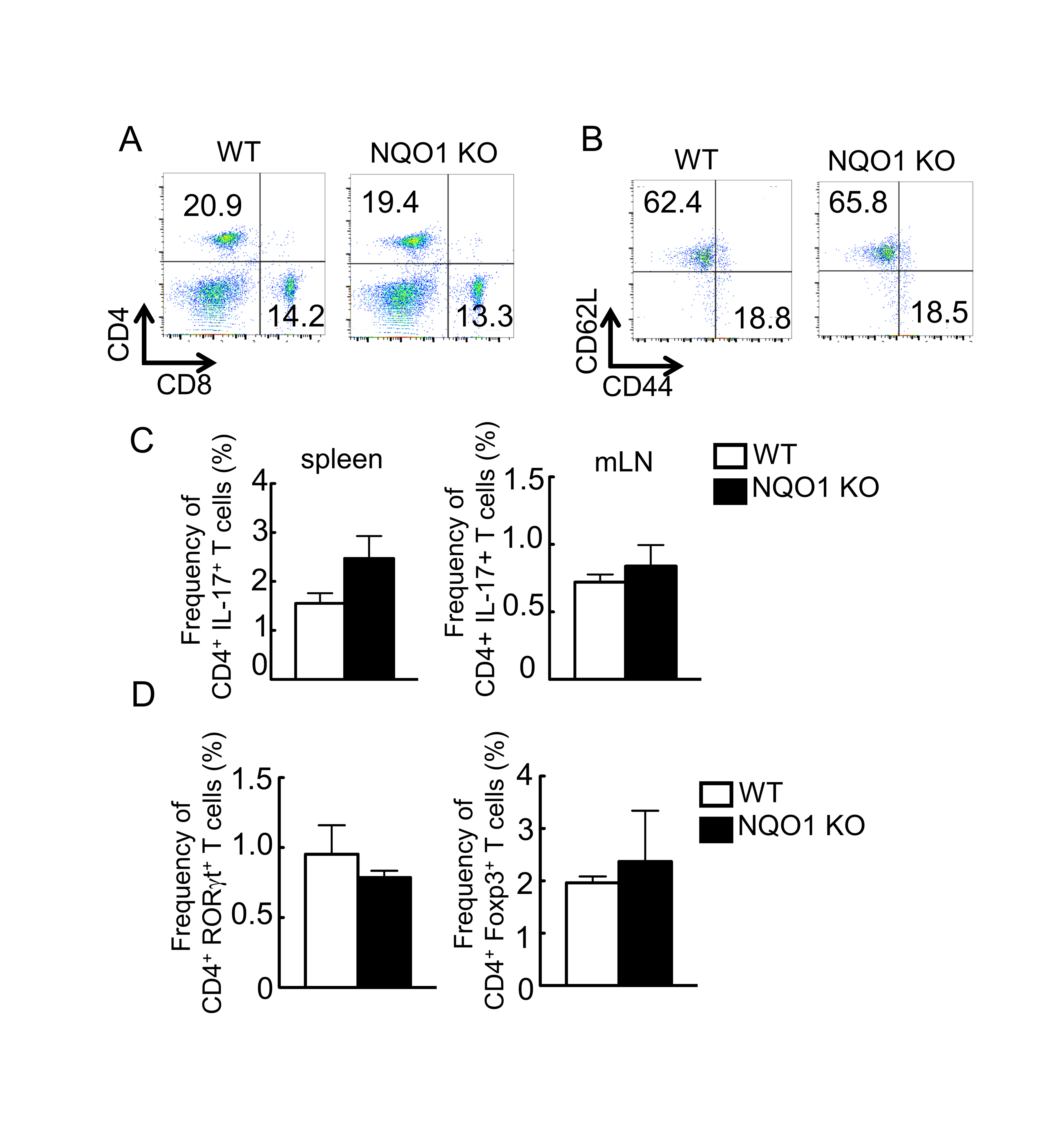

Supplement: S2 Fig — (A) Splenocytes from Nqo1-/- and WT mice were stained for CD4 and CD8. The numbers indicated the percentages of cells (n = 4). (B) The gated CD4+ T cells were analyzed CD44 and CD62L expression in splenocytes (n = 4). (C) Splenocytes and lymphocytes from Nqo1KO and WT mice were stimulated with PMA, Ionomycin and BFA for 4 h and analyzed for IL-17A expressing CD4+ T cells by flow cytometry. Right panel showed the percentages of IL-17+ CD4+ T cells in mesenteric lymph node, and left panel displayed it in spleen from Nqo1 KO and WT mice (n = 4). (D) The frequency of CD4+ RORγt+ T cells or CD4+ Foxp3+ T cells in splenocytes from Nqo1 KO and WT mice in steady state (n = 4). These graphs indicate the mean ± SE, Unpaired T-test. (TIF) [file pone.0272090.s002.tif]

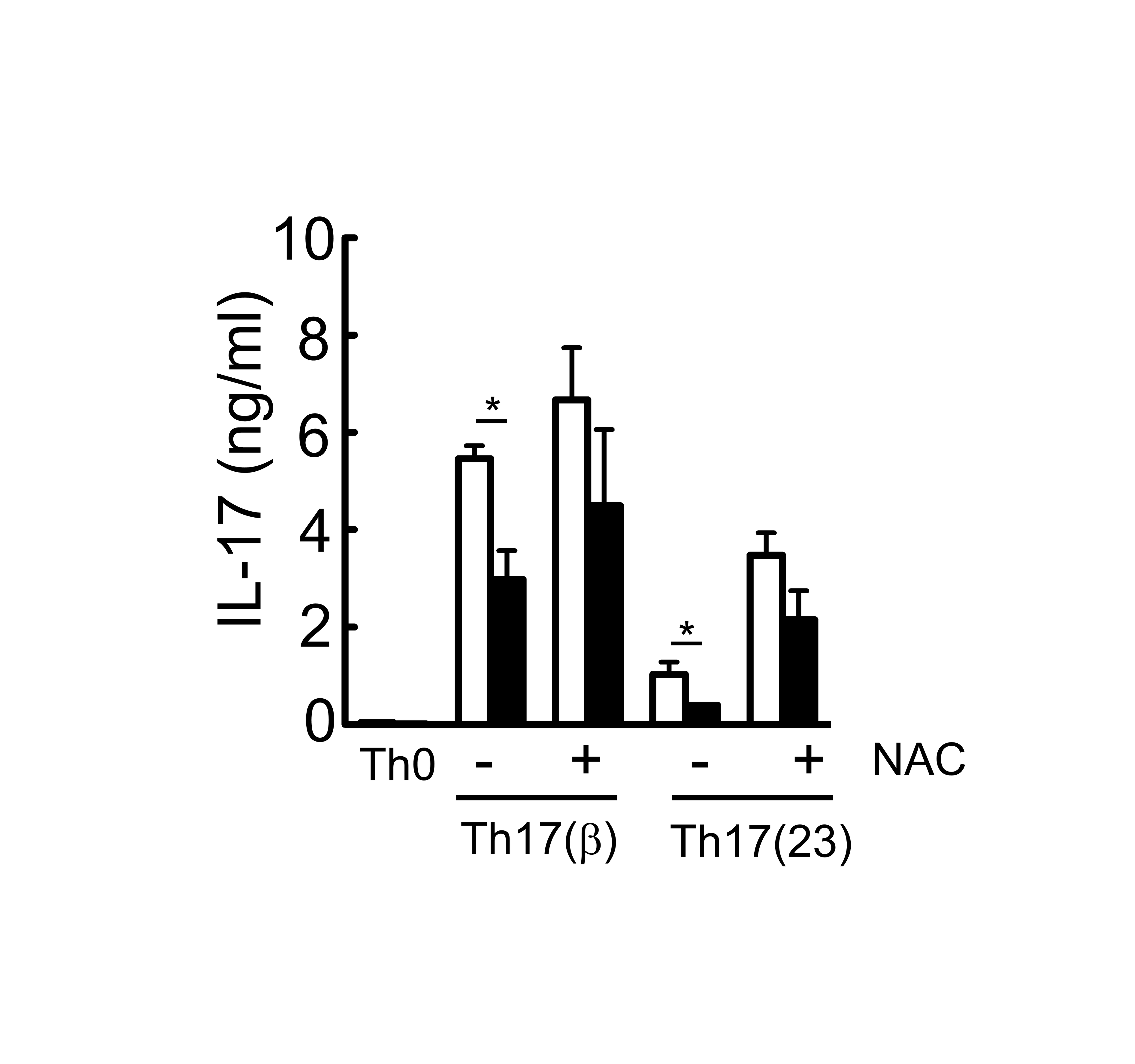

Supplement: S3 Fig — Naïve CD4+ T cells from WT and Nqo1-deficient mice were cultured under the Th17 conditions media for 72 h with or without 5 mM NAC. IL-17A production was measured by ELISA (n = 4). This graph indicates the mean ± SE, One way ANOVA. (TIF) [file pone.0272090.s003.tif]

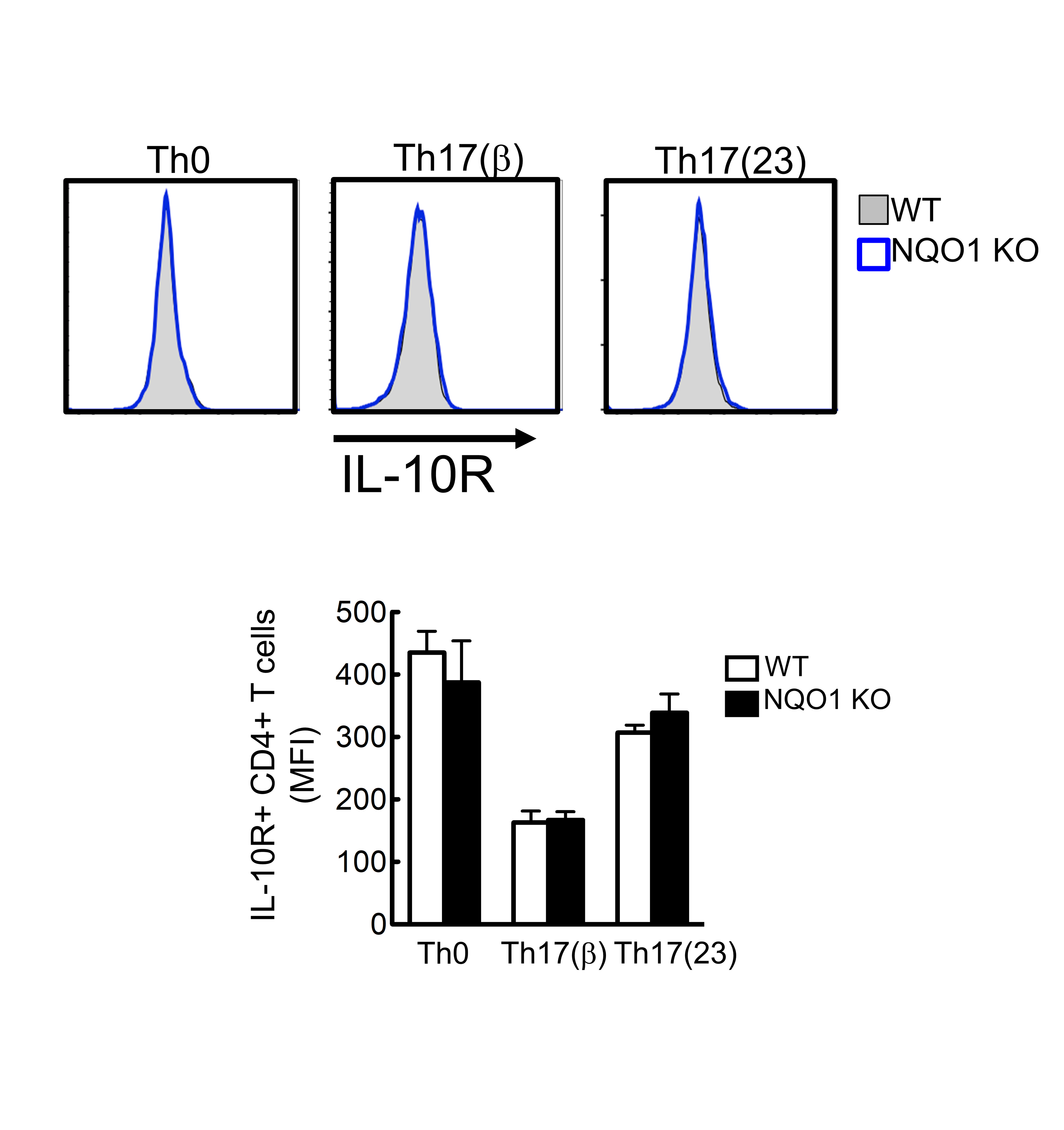

Supplement: S4 Fig — Naïve CD4+ T cells from Nqo1 deficiency and WT mice were cultured under the Th17 conditions media for 48 h. The graph showed expression of IL-10 receptor α (n = 4). This graph indicates the mean ± SE, One way ANOVA. (TIF) [file pone.0272090.s004.tif]
